# Vertebrate pheromones and other semiochemicals: the potential for accommodating complexity in signalling by volatile compounds for vertebrate management

**DOI:** 10.1042/BST20140134

**Published:** 2014-08-11

**Authors:** John A. Pickett, Stephen Barasa, Michael A. Birkett

**Affiliations:** * Rothamsted Research, Harpenden, Hertfordshire AL5 2JQ, U.K.

**Keywords:** attractant, pest control, pheromone, repellent, semiochemical, signalling, vertebrate

## Abstract

The interaction between volatile and non-volatile, e.g. proteinaceous, components of pheromone and other semiochemical-based signalling systems presents a daunting set of problems for exploitation in the management of vertebrates, good or bad. Aggravating this is the complexity of the mixtures involved with pheromones, not only by definition associated with each species, but also with individual members of that species and their positions within their immediate communities. Nonetheless, already in some contexts, particularly where signals are perceived at other trophic levels from those of the vertebrates, e.g. by arthropods, reductionist approaches can be applied whereby the integrity of complex volatile mixtures is maintained, but perturbed by augmentation with individual components. In the present article, this is illustrated for cattle husbandry, fish farming and human health. So far, crude formulations have been used to imitate volatile semiochemical interactions with non-volatile components, but new approaches must be developed to accommodate more sophisticated interactions and not least the activities of the non-volatile, particularly proteinaceous components, currently being deduced.

## Introduction

All organisms exploit some form of chemical communication via signals termed semiochemicals, e.g. pheromones, which are involved in communication within species. For vertebrates, other modalities for communication appear to eclipse the role of semiochemicals in determining life-important behaviour, but closer examination reveals essential contributions from semiochemicals associated with mating, social ranking and food location/acceptability. This is particularly true of mice, *Mus musculus domesticus* [1]. Together with other rodents such as rats, mice present an extremely serious social and economic problem [2], and new approaches to their control are avidly researched. Because of the complexity of the pheromonal system of mice, practical use of already identified chemistry has been marginal. In the present review, we concentrate on the volatile components of the pheromonal systems, with lessons of practical use drawn only from semiochemical-based control of insects and other arthropods that cause direct damage to, or act as vectors of pathogens for, ourselves and other vertebrates, principally farmed animals. However, because of the involvement of non-volatile, i.e. proteinaceous, components in mouse pheromonal communication, the simple fixative formulations currently employed for arthropod semiochemical release are expected to be replaced with synthetic protein pheromone components with pheromonal functions that create a molecular structure-based release profile.

As with control of arthropod pests of vertebrates, control of rodents with toxicants is failing mainly because of selection for toxicant resistance. Similar to the situation for arthropod pests, there is a long history of using elements of semiochemical communication in rodent control, although, for the latter, this is largely restricted to food-related baits. For arthropods, other types of semiochemicals have been used for control, including attractant pheromones and repellents for individual protection, rather than general population reduction provided by lure and kill technologies. The ultimate goal of current work (BBSRC grant BB/J001821/1, The interplay of rodent behaviour and semiochemistry: from scientific principles to control strategies) is to develop monitoring of populations based on attractancy and then control by combining this with repellency in a push–pull (stimulo–deterrent diversionary) strategy, as has been successfully developed for pest management in crop-based agriculture [3]. Thus, after identifying appropriate push and pull semiochemicals using new methodologies devised for the arthropods, rodents would be pushed from sensitive regions and pulled to sites for capture and removal, by selective toxicants or pathogen delivery, or by other means of elimination. For cost effectiveness and environmental considerations, the biochemical routes by which the semiochemicals are produced would need to be exploited. Higher plants, perhaps after genetic modification, could then be used to produce volatile semiochemicals, and overexpression of genes for protein pheromone components and as slow release substrates would need to be employed after expression by GM (genetically modified) fermentation organisms. Registration of the semiochemical-based systems would be required, but would be facilitated by the use of vertebrate-compatible nature-identical agents acting by non-toxic mechanisms. There will be selection for resistance to these systems where used excessively, but these will involve multigenic mechanisms, as opposed to the often single non-synonymous SNPs (single nucleotide polymorphisms) involved in toxicant resistance, and will involve, by analogy with arthropod resistance to semiochemicals [4,5], selection of alternative semiochemical components to provide the original signal, the use of which is likely not to change as it comprises an essential component of the rodent behavioural ecology. The new semiochemicals necessary for overcoming resistance would be identified as for the original compounds and probably occur already, and thereby would have been identified previously, but as components with only minor behavioural contribution.

## Attractant semiochemicals

**Figure 1:**
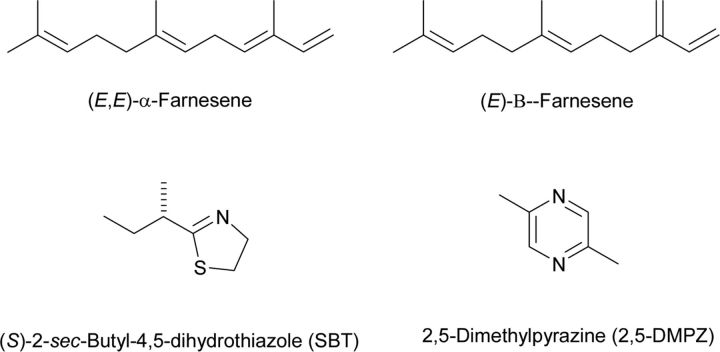
Potentially positive-acting volatile semiochemicals for mouse urine

**Figure 2:**
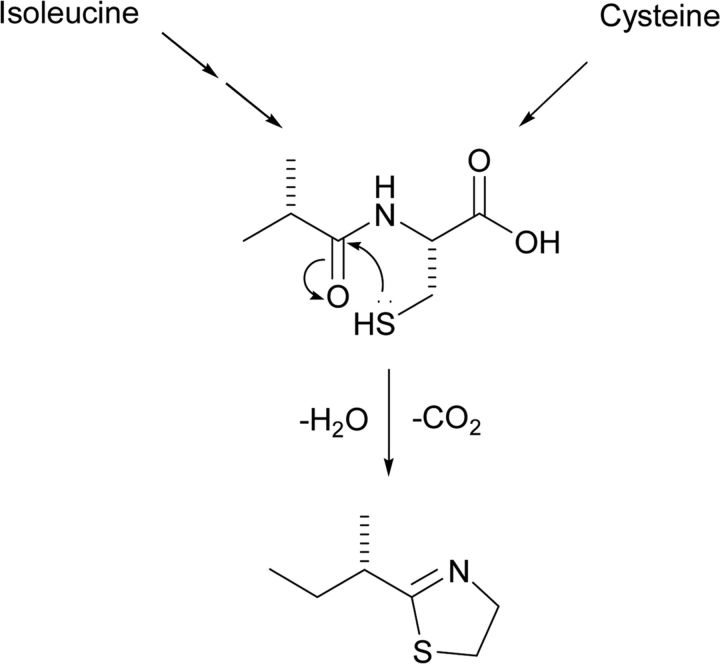
Proposed biosynthesis of the mouse volatile compound SBT

Attractant semiochemicals act as signals perceived by the sensory system and without physiological effects, and cause oriented movement towards the origin. Arising from food or food baits, this is an obvious process and has been exploited already for rodents. However, many other aspects of rodent ecology offer the potential for capturing semiochemicals that relate to positive behaviours inducing attraction. For the mouse, Figure 1 shows potentially positively acting volatile semiochemicals associated with mouse urine. The specific roles are complicated by contextual issues relating to sex and hierarchical positions within the mouse community [6]. The biosynthesis of these compounds represents a number of biosynthetic pathways that must relate to mouse genetics, so far mostly not annotated in the mouse genome [7], with identification likely to be facilitated via the transcriptome [e.g. NGS (next-generation sequencing) or RNA-Seq (RNA sequencing)] associated with the presence of these compounds in the urine. For SBT [(*S*)-2-*sec*-butyl-4,5-dihydrothiazole], the proposed route involving oxidation of isoleucine before cysteine conjugation can be presumed to involve a cytochrome P450 enzyme (Figure 2). Other positively acting semiochemicals are associated with the body of the mouse, but may have their origins in exocrine secretions from various organs, e.g. those associated with mucus-producing regions [8]. Although it is by no means clear how the known compounds could be used in pull systems for mice with much new work needing to be done, such semiochemicals are already being used routinely for arthropod control, e.g. the tsetse fly *Glossina morsitans*, the vector of *Trypanosoma brucei*, which is the causative agent of nagana in cattle in Africa, is pulled into traps baited with host urine from the buffalo S*yncerus kafir* and acetone, a component of host breath. The traps incorporate blue textile, and destruction of the flies is by desiccation or control using black material impregnated with the Rothamsted-invented pyrethroid deltamethrin [9]. The urine itself, as an alternative source of semiochemicals, is appropriate for input-limited farmers and pastoralists, but a completely chemical host lure (POCA) is available comprising 3-*n*-propylphenol, 1-octen-3-ol, 4-methylphenol (*p*-cresol) and acetone [10]. This approach is currently being developed for control of other tsetse flies, e.g. *Glossina palpalis*, that are vectors for trypanosomes causing HAT (human African trypanosomiasis) [11], but more specific host cues may need to be identified and the component 1-octen-3-ol provided as a single isomer as for attraction of *Culicoides* spp. biting midges [12].

## Repellent semiochemicals

**Figure 3:**
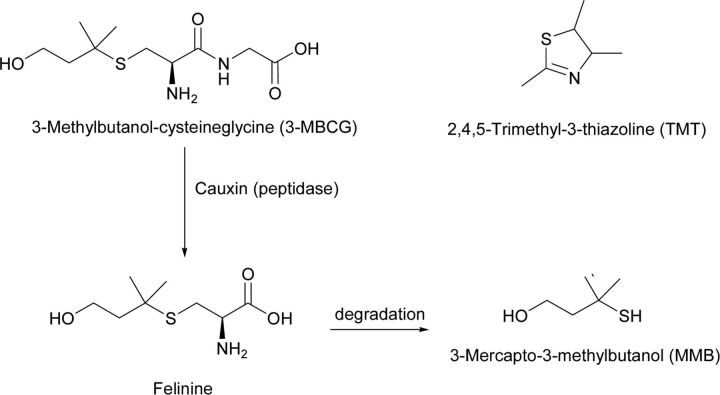
Volatile compounds from predator cats (felinine) and red fox (TMT)

Behavioural aversion, as a consequence of sensory perception of semiochemicals indicating a disadvantage to the recipient, is associated with inappropriate food and animals that are unsuitable as hosts. For rodents, semiochemicals derived from predators have already been studied, but insufficient knowledge is currently available for these to be used as push components of the push–pull strategy. Figure 3 includes some of the chemicals involved for the cat *Felis domesticus* [13] and the red fox *Vulpes vulpes* [14], again with unknown genetics in the emitting animal.

The range of repellents can be defined ecologically, and, for better understanding, should be separated from toxicants e.g. DDT [1,1,1-trichloro-2,2-bis-(*p*-chlorophenyl)ethane] and volatile pyrethroids such as metofluthrin, which appear to cause a sublethal aversion rather than a truly behavioural repellent effect. Behavioural repellents can be ranked according to their role in the ecology of the animal being repelled and have been best defined for arthropods attacking vertebrates [15,16]. The most widely commercially developed botanically derived repellents are thought to signal an inappropriate ecosystem for finding a vertebrate-derived blood meal. Traditionally, essential oils such as citronella from lemongrass, *Cymbopogon* spp., have been used, with more recently specific essential oil components being targeted, e.g. sesquiterpenes or the iridoid isoprenoids such as isomers of nepetalactone [17,18]. The essential oil components that cause true repellency appear to be detected by specific olfactory neurons in carnivorous insects such as mosquitoes, including vectors of pathogens causing malaria and Dengue fever, i.e. *Anopheles gambiae* sensu stricto and *Aedes aegypti* respectively. These same cells can, although they are less sensitive, respond to commercial repellents such as DEET (*N,N*-diethyltoluamide) [19–21] and other products more recently developed by structure–activity relationship studies, e.g. picaridine.

The next level of repellency is derived for semiochemicals from species close to the host taxa. From work at the *icipe* (International Centre of Insect Physiology and Ecology), based in Kenya and including unpublished work (R. Saini, personal communication) on the control of tsetse flies, repellents have been developed from the waterbuck *Kobus defassa.* Although closely related to the preferred host species, this member of the Bovidae is not attacked by *G. morsitans* and the repellent compounds have been identified [22,23]. A similar example is an arthropod that attacks salmonid fish, the salmon louse *Lepeophtheirus salmonis*, which is highly developed at the free swimming copopodid stage in locating and attaching to host salmonids, but avoids turbot, *Scophthalmus maximus* [24–26]. In three seasons, fish farming pens holding salmon had significantly reduced levels of fish lice when protected with a polymeric rope providing a slow release of the single turbot component 2-aminoacetophenone [27].

The final classification for repellents is those from within the host species, but from unattractive individuals. This was first elucidated for flies attacking cattle, and tested the hypothesis that those individuals within a herd of a single breed with consistently low fly load produced more of, or additional, compounds that interfere with the normal attractiveness of the species [28]. Indeed, just one of these semiochemicals, 6-methyl-5-hepten-2-one, emanating from a polymeric slow-release substrate positioned on the cow's back, reduced the fly load of a normally high loaded cow to that of an individual normally with the lowest fly loading [29]. This is being exploited in a breeding programme based on the identification of the regulatory and functional genes associated with production of compounds, including 6-methyl-5-hepten-2-one, that reduce attractiveness of cattle to arthropods (BBSRC grant BB/K007610/1, Defining the genetic and semiochemical basis of tick resistance in cattle). The identification of functional genes is facilitated again by the postulated biosynthetic route for 6-methyl-5-hepten-2-one by oxidative cleavage of steroidal side chain by a cytochrome P450 already generically annotated in the cow genome [30]. Analogous work on human-derived semiochemicals has allowed identification, using coupled GC–electrophysiology, of compounds from individuals unattractive to mosquitoes and the Scottish biting midge *Culicoides impunctatus* as comprising 6-methyl-5-hepten-2-one together with (*E*)-6,10-dimethyl-5,9-undecadien-2-one and long-chain aldehydes [31]. We have evidence that the longer-chain ketone derives from oxidation of squalene produced in human skin sebocytes (M.A. Birkett, unpublished work), and the genetic basis of this semiochemically based unattractiveness is under investigation using human twins (J. Armour and J. Logan, personal communication). The chemistry is patented and being developed as a contextual repellent for use in protecting individual human subjects, with considerable promise in field trials where it appears to be as highly effective as DEET, although less persistent as a consequence of higher volatility [32].

## Push–pull system

The prospect of utilizing push–pull systems for control of pests affecting vertebrates has already been reported [33–35]. *icipe* is establishing a push–pull system for the tsetse fly *G*. *morsitans* with extensive support from the European Union. The visual traps with urine and acetone and the killing agent deltamethrin provides the pull, whereas the push comprises a collar around the cow's neck, slowly releasing a mixture of waterbuck non-host contextual repellents, with results thus far being extremely promising.

## Conclusion

There is ample evidence from related animal interactions for the use of semiochemicals in management of arthropods attacking vertebrates. Although substantial work is needed to realize these opportunities for rodent management and particularly mice and rats, it is proposed that a syntheses of the knowledge gained so far could produce the essence of a push–pull strategy for control. Although in some ways a complication, the equally important role of non-volatile proteinaceous pheromonal components could help us to develop the syntheses by providing a novel means of selective molecular-based release of the volatile components.

## Abbreviations

DEET: *N*,*N*-diethyltoluamide
*icipe*: International Centre of Insect Physiology and Ecology
SBT: (*S*)-2-*sec*-butyl-4,5-dihydrothiazole
